# Bursopentin (BP5) Protects Dendritic Cells from Lipopolysaccharide-Induced Oxidative Stress for Immunosuppression

**DOI:** 10.1371/journal.pone.0117477

**Published:** 2015-02-06

**Authors:** Tao Qin, Yinyan Yin, Qinghua Yu, Qian Yang

**Affiliations:** Key Lab of Animal Physiology and Biochemistry of China’s Department of Agriculture, College of Veterinary Medicine, Nanjing Agricultural University, Nanjing, People’s Republic of China; Universidade de São Paulo, BRAZIL

## Abstract

Dendritic cells (DCs) play a vital role in the regulation of immune-mediated inflammatory diseases. Thus, DCs have been regarded as a major target for the development of immunomodulators. However, oxidative stress could disturb inflammatory regulation in DCs. Here, we examined the effect of bursopentine (BP5), a novel pentapeptide isolated from chicken bursa of fabricius, on the protection of DCs against oxidative stress for immunosuppression. BP5 showed potent protective effects against the lipopolysaccharide (LPS)-induced oxidative stress in DCs, including nitric oxide, reactive oxygen species and lipid peroxidation. Furthermore, BP5 elevated the level of cellular reductive status through increasing the reduced glutathione (GSH) and the GSH/GSSG ratio. Concomitant with these, the activities of several antioxidative redox enzymes, including glutathione peroxidase (GPx), catalase (CAT) and superoxide dismutase (SOD), were obviously enhanced. BP5 also suppressed submucosal DC maturation in the LPS-stimulated intestinal epithelial cells (ECs)/DCs coculture system. Finally, we found that heme oxygenase 1 (HO-1) was remarkably upregulated by BP5 in the LPS-induced DCs, and played an important role in the suppression of oxidative stress and DC maturation. These results suggested that BP5 could protect the LPS-activated DCs against oxidative stress and have potential applications in DC-related inflammatory responses.

## Introduction

Dendritic cells (DCs) play a key role in the immune system, bridging the innate and adaptive immune responses [1]. As professional antigen-presenting cells (APCs), they are able to capture, process, and present the antigens to naïve T cells [2], as well as regulate the inflammatory response through the secretion of cytokines and chemokines [3,4]. However, under certain conditions, activation of DCs may instead contribute to disease pathogenesis [5]. Prominent roles have been observed in diseases such as autoimmunity and chronic inflammation, rendering DCs an attractive target for therapeutic intervention [6–9].

Oxidative stress has been shown to play an important role in a wide range of immune responses [10]. In conventional DCs, oxidative stress acts as a regulator of both inflammatory responses and the polarisation of T cells [11–13]. For example, the allostimulatory ability of DCs is affected directly by glutathione levels, in that glutathione depletion is sufficient to inhibit DC function, and this function can be rescued by reconstitution of glutathione [13]. In Kupffer cells, the primary APCs in the liver, reactive oxygen species (ROS) serve as essential mediators of antigen presentation [14]. In contrast, deleterious effects have been demonstrated in several autoimmune and inflammatory diseases, including type 1 diabetes, asthma, and inflammatory bowel disease (IBD) [15–17], in part through the regulation of inflammatory responses. In the pathogenesis of type 1 diabetes, ROS not only cause direct damage to β cells, but also participate actively in inflammation [18]. Control of oxidative stress can be achieved through a variety of means, including dietary antioxidants, such as β-carotene [19], vitamin C [20], lycopene [21], and a combination of vitamins C and E [22], or through pharmaceutical intervention, using anti-inflammatory drugs such as dextromethorphan [23], panax quinquefolium saponins [24], and fisetin [25]. As many of these compounds have been shown to exert anti-inflammatory effects by inhibiting the activation and maturation of DCs, the use of these or other antioxidants may be useful for the management of inflammatory diseases associated with DC activity.

The bursa of fabricius (BF), the central humoral immune organ unique to birds, plays a critical role in the development of the B cell compartment [26,27]. The BF therefore provides a valuable model for studying basic physiology and immunology in mammals. Recently, a variety of small bioactive peptides have been isolated from the BF. Bursin, the first peptide isolated from the BF, can induce the differentiation of B cells [28,29]; other peptides, including BSP-II [30], BPP-II [31], BP-I to III [32], and BP11 [33], have exhibited other immunomodulatory roles. Among these peptides, bursopentine (BP5, with an amino acid sequence of Cys-Lys-Asp-Val-Tyr) has drawn significant interest due to its multi-functional modulation of immune responses. BP5 not only affected hybridoma cells and B and T lymphocytes [34,35], but it also protected macrophages against oxidative stress [36]. Recent work by our laboratory has shown that BP5 can inhibit a wide range of DC functions, including phenotypic maturation and pro-inflammatory cytokine secretion, migration, and allostimulatory abilities [37].

Here, we have expanded on this work, characterizing the antioxidative effects of BP5 in DCs and the relationship between BP5 and the immune function of DCs in the presence of intestinal epithelial barriers. Taken together, these data suggest that BP5 may provide a simple, inexpensive, and highly effective strategy for regulating DC activity in chronic inflammatory diseases.

## Materials and Methods

### Ethics statement

The Animal Ethics Committee at Nanjing Agricultural University reviewed the protocol and approved this study specifically, with the project number 2009ZX08009-138B. The slaughter and sampling procedures strictly followed the guidelines on Ethical Treatment of Experimental Animals (2006) No. 398 set by the Ministry of Science and Technology, China and the Regulation regarding the Management and Treatment of Experimental Animals (2008) No. 45 set by the Jiangsu Provincial People’s Government. C57BL/6 mice (4–6 weeks old) were purchased from the Animal Research Center of Yangzhou University (Yangzhou, China). All efforts were made to minimize the number of animals used and their suffering. The animals were acclimatized for 1 week before the study and had free access to water and standard mice chow throughout the experiment. Mice were euthanized with inhalation of 100% CO_2_.

### Reagents

Fetal bovine serum (FBS) was purchased from Hyclone (Thermo, Melbourne, Australia). RPMI1640 medium (no phenol red), penicillin and streptomycin were purchased from Invitrogen (Grand Island, NY, USA). Recombinant GM-CSF and IL-4 were from Peprotech (Rocky Hill, NJ). Lipopolysaccharide (LPS) derived from *Escherichia coli* 026:B6 was from Sigma (St. Louis, MO). FITC-CD80, PE-CD86, FITC-MHCII or respective isotypes were from eBioscience (San Diego, CA, USA). Rabbit anti-mouse heme oxygenase 1(HO-1), rabbit anti-mouse β-actin and goat anti-rabbit IgG-horseradish peroxidase (HRP) were from Bioworld (St. Louis Park, MN, USA). Micro BCA protein assay kit was from Pierce (Rockford, IL). Cobalt protoporphyrin (CoPP), an inducer of HO-1 [38], and tin protoporphyrin IX (SnPP), an inhibitor of HO-1 activity [38], were from Sigma. Synthetic BP5 was purchased from Biotech Bioscience and Technology Co., Ltd (Shanghai, China). The purity of the synthetic peptide was > 98% by RP-HPLC. The sequence of the synthetic peptide was examined by electrospray ionisation tandem mass spectrometry (ESI-MS/MS). It was also tested the LPS contamination using the E-Toxate Limulus LPS detection kit (Sigma), which is sensitive to 0.05 to 0.1 endotoxin units/ml. Only uncontaminated preparations were used.

### Generation of DCs

DCs were isolated and cultured as our improved method [37]. Briefly, bone marrow cells were flushed from the tibias and femurs and cultured in complete medium (RPMI1640 with 10% FBS, 1% streptomycin and penicillin, 10 ng/ml GM-CSF and IL-4). On day 3, medium was gently discarded and fresh medium was added. On day 6, non-adherent and loosely adherent DC aggregates were harvested and subcultured overnight. On day 7, 90% or more of the CD11c^+^ non-adherent cells were used. In our previous study, we confirmed the nontoxic concentration of BP5 (0.5–200 μg/ml) and LPS (10 ng/ml-1 μg/ml) [37].

### Detection of nitric oxide (NO) production

As an indicator of NO synthesis, the concentrations of nitrite (NO_2_^−^) in the DCs culture medium were estimated using the Griess reagent[39]. A NO detection kit (Beyotime, China) was used according to the manufacturer’s protocol. Briefly, the 50 μl supernatant was mixed with an equal volume of Griess reagents I and II at room temperature. The absorbance was measured at 540 nm. The NO production was calculated from a standard curve sodium nitrite (NaNO_2_).

### Determination of ROS generation

Intracellular ROS levels were determined by measuring the oxidative conversion of cell permeable 2’, 7’ dichlorofluorescein diacetate (DCFH-DA) to fluorescent dichlorofluorescein (DCF). Briefly, after collecting cells from different groups, DCs (1×10^6^ cells/ml) were incubated with 10 μM DCFH-DA at 37°C for 20 min. In the positive control, the DCFH-DA-loaded cells were then treated with 50 μM H_2_O_2_ for 20 min. The fluorescent product DCF was determined at 485 nm excitation and 530 nm emission using a fluorescence microplate reader (Bio-TEK, USA). The fluorescent cells also were detected by flow cytometry (FACS).

### Determination of lipid peroxidation

Maleic dialdehyde (MDA), a reliable marker of lipid peroxidation, was determined with thiobarbituric acid (TBA) according to the manufacturer’s instruction (NJBC, Nanjing, China). 100 μl of cell lysate supernatant was mixed with 1 ml TBA working solution. Each reaction mixture was heated for 40 min at 95° and cooled to room temperature. After centrifugation at 1,200 g for 10 min, the organic layer was collected and the absorbance was measured at 530 nm.

### Measurement of the reduced glutathione (GSH) and the oxidized glutathione (GSSG)

Cell extracts were prepared by sonication in ice-cold 5% metaphosphoric acid and centrifuged at 10,000 g for 20 min to remove debris, and the supernatant fluid was collected. Then GSH content and total glutathione/GSSG in the supernatant were determined by GSH and T-GSH/GSSG kits, respectively (NJBC, Nanjing, China). The absorbance of the resulting yellow color was measured using a spectrophotometer at 420 nm. To calculate the GSH/GSSG ratio, GSH (reduced form) was obtained by subtracting the 2×GSSG values from the total glutathione values.

### Assay of antioxidant enzyme activity

The glutathione peroxidase (GPx) activity was assayed with commercial GPx assay kit (Beyotime, China). Briefly, 10 μl lysate was mixed with 10 μl GPx assay working solution (10 mM NADPH, 84 mM GSH, and glutathione reductase solution), 176 μl GPx assay buffer at 25°C for 5 min. Reactions were initiated by 4 μl of 15 mM cumene hydroperoxide, and absorbance was measured at 340 nm for 3 min. The blank and background control were also set.

Superoxide dismutase (SOD) activity in the lysate of DCs was quantified with a kit (NJBC, Nanjing, China). The absorbance was measured at 450 nm using a spectrophotometer.

For the catalase (CAT) activity, CAT assay kit (Beyotime, China) was used in accordance with the manufacturer’s instruction. Briefly, the lysate of cells was treated with excess H_2_O_2_ for decomposition by catalase for an exact time, and the remaining H_2_O_2_ coupled with a substrate, was treated with peroxidase to form a red product, N-4-antipyryl-3-chloro-5-sulfonate-p-benzoquinonemonoimine, which can be read at 520 nm of absorbance. Thus, CAT activity can be assessed by measuring the decomposition of H_2_O_2_.

### Establishment of the ECs/DCs coculture system

ECs/DCs coculture system were established as previously described with some modifications [40]. Caco-2 epithelial cells (ATCC) were cultured on the upper side of membrane inserts (pore size, 3 μm; Greiner Bio-One, Germany) in a 24 well plate. The cells were maintained until the transepithelial electric resistance (TEER) of ~ 300 ohm×cm^2^. Filters were turned upside down and then DCs (5×10^5^) were seeded facing the basolateral side of Caco-2 cells for 4 h to let them attach to the filter. Then the filters were turned upside-down again into 24-well plate.

### Confocal microscopy

The fixed filters were permeabilized in 0.2% Triton X-100 for 5 min and blocked with 5% BSA for 1 h, and then were labeled with primary antibodies overnight at 4°C followed by fluorescent secondary antibodies for 1 h at room temperature. The filters were observed by confocal laser scanning microscopy (CLSM) (LSM 710, Zeiss, Oberkochen, Germany). Serial optical sections were collected by Z-project model with a 0.5 μm increment on the z-axis.

### Phenotype and cytokine assay

DCs were harvested and incubated with FITC-CD80, FITC-MHCII, PE-CD86 or the respective isotypes at 4°C for 30 min as per manufacturer’s guidelines. After washing three times, DCs were detected by FACS. Supernatants were collected and the expression of TNF-α was detected by ELISA using commercial immunoassay kit (Boster, Wuhan, China) in accordance with the manufacturer’s instruction.

### Western blot analysis

DCs were lysed with RIPA buffer (Beyotime, China), including protease inhibitor. The lysate was centrifuged at 12,000 g for 10 min at 4°C and the protein concentrations were determined. Protein extracts were resolved on SDS-polyacrylamide gels, transferred to polyvinylidene fluoride membranes, blocked with phosphate-buffered saline (PBS) containing 0.05% Tween (PBST) and 5% dry powdered milk, and probed with antibodies specific for HO-1 and β-actin. Signals were detected with HRP-labeled secondary antibodies by using chemiluminescence labeling. Autoradiograms were scanned and analyzed with Quantity One (Bio-Rad, Hercules, CA) to quantify band densities.

### Statistical analysis

Results were expressed as the means ± SD. Statistical significance was determined by one way analysis of variance (ANOVA) followed by Dunnett’s t-test to evaluate variations between groups with value of *P* < 0.05 considered to be statistically significant.

## Results

### BP5 inhibited the production of NO in the LPS-stimulated DCs

**Fig 1:**
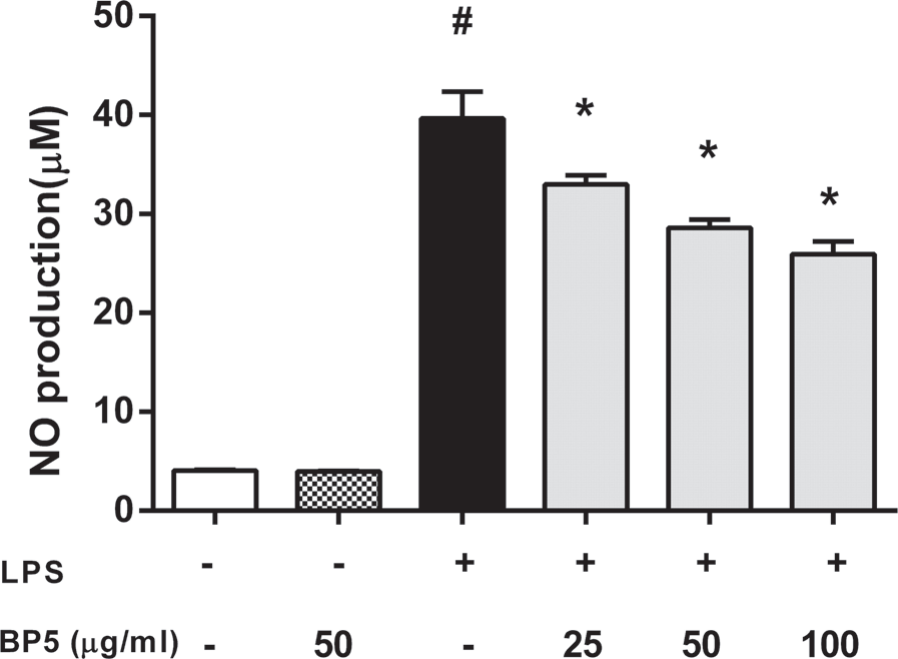
BP5 efficiently suppressed NO production in DCs. DCs (1×10^6^ cells/ml) were incubated with the indicated concentrations of BP5 for 2 h, and then incubated with or without LPS (100 ng/ml) for 22 h. Supernatants were collected and NO production was measured using the Griess reagent. Data shown are the means ± SD of three samples. *P < 0.05 in comparison with the LPS-only group; ^#^ *P* < 0.05 in comparison with the untreated group. All results are representative of three independent experiments.

NO has an established role in the defense against bacterial infections, and exerts multiple modulatory activities on both inflammation and immune responses [41]. We examined the inhibitory effect of BP5 on NO by LPS-stimulated DCs. LPS significantly increased NO production, compared with control. However, BP5 remarkably attenuated the LPS-induced NO production (Fig. 1).

### BP5 inhibited intracellular ROS generation in the LPS-stimulated DCs

**Fig 2:**
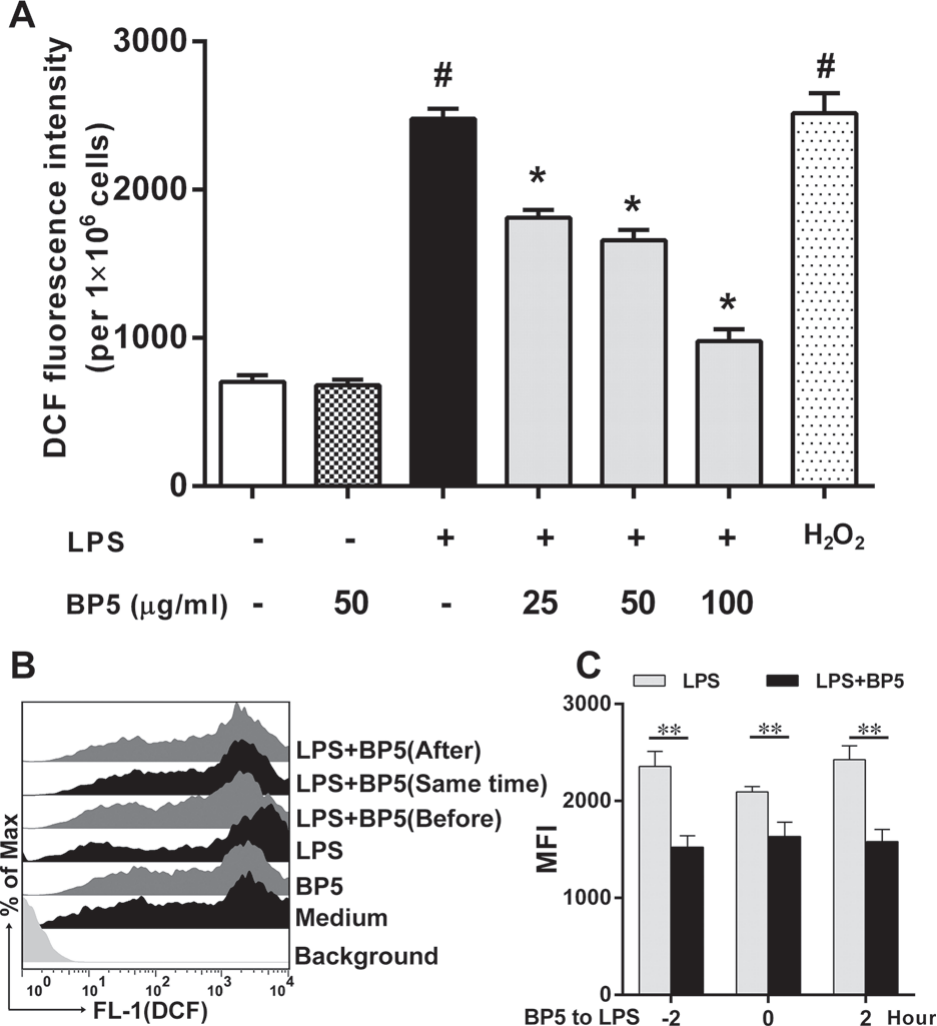
BP5 impaired the production of ROS by LPS-stimulated DCs. (A) DCs (1×10^6^ cells/ml) were incubated with the indicated concentrations of BP5 for 2 h, and then incubated with or without LPS (100 ng/ml) for 22 h, and then incubated with 10 μM DCFH-DA at 37°C for 20 min. ROS production was detected by fluorescence microplate reader. H_2_O_2_ (50 μM) was used as a positive control. (B-C) DCs stimulated with LPS before, at the same time as, or after BP5 (50 μg/ml) incubation. ROS production was detected by FACS. Data shown are the means ± SD of three samples. **P < 0.01, *P < 0.05 in comparison with the LPS-only group; ^#^ *P* < 0.05 in comparison with the untreated group. All results are representative of three independent experiments.

We assessed the intracellular ROS as the response of the stimulated DCs. The generation of ROS was determined by monitoring a conversion of DCFH_2_-DA to DCF. After exposure to LPS for 22 h, ROS production was increased significantly, while it was effectively inhibited by BP5. As a positive control, DCs exposed to H_2_O_2_ (Beyotime, China) also exhibited a significant increase over the control (Fig. 2A). Furthermore, using the FACS, ROS production was decreased in all tests, regardless of whether BP5 treatment was administered to DCs before, at the same time as, or after LPS stimulation (Fig. 2B, C).

### BP5 suppressed lipid peroxidation in the LPS-stimulated DCs

**Fig 3:**
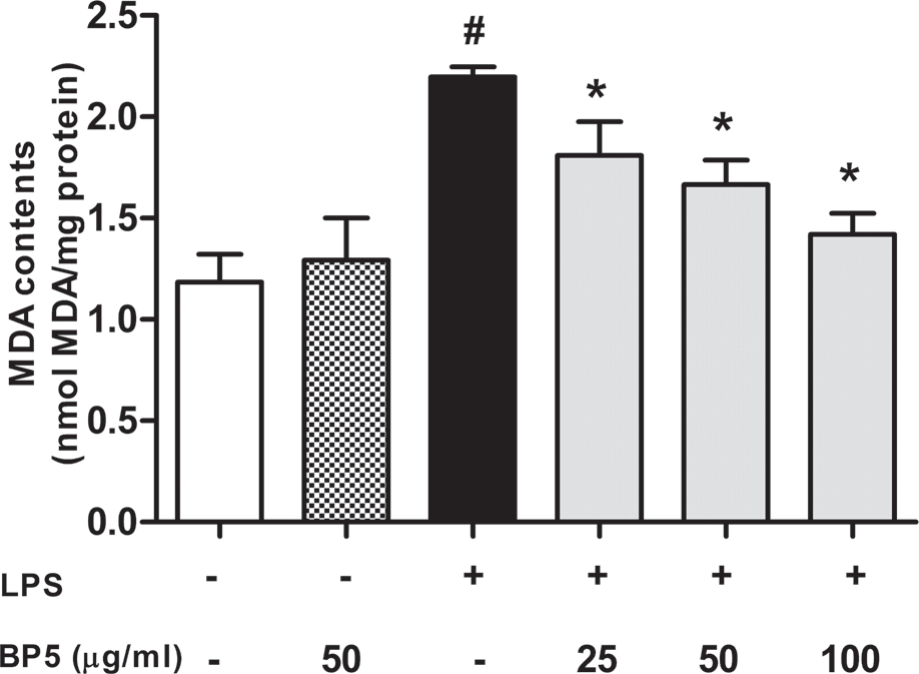
BP5 suppressed lipid peroxidation in the LPS-stimulated DCs. DCs (1×10^6^ cells/ml) were pretreated with or without BP5 for 2 h, and then exposed to LPS (100 ng/ml) or not for 22 h. The MDA contents in cell lysate supernatants were measured as described in Materials and Methods. Data shown are the means ± SD of three samples. *P < 0.05 in comparison with the LPS-only group; ^#^ *P* < 0.05 in comparison with the untreated group. All results are representative of three independent experiments.

To determine whether BP5 had anti-lipid peroxidation activities in DCs, the intracellular level of malondialdehyde (MDA) was measured. MDA, one of the final products of polyunsaturated fatty acid peroxidation, is a common marker of oxidative stress [50]. As shown in Fig. 3, MDA concentrations were elevated significantly in the LPS-treated cells. However, BP5 remarkably suppressed this process.

### BP5 modulated intracellular GSH, GSSG, and the GSH/GSSG ratio in the LPS-stimulated DCs

**Fig 4:**
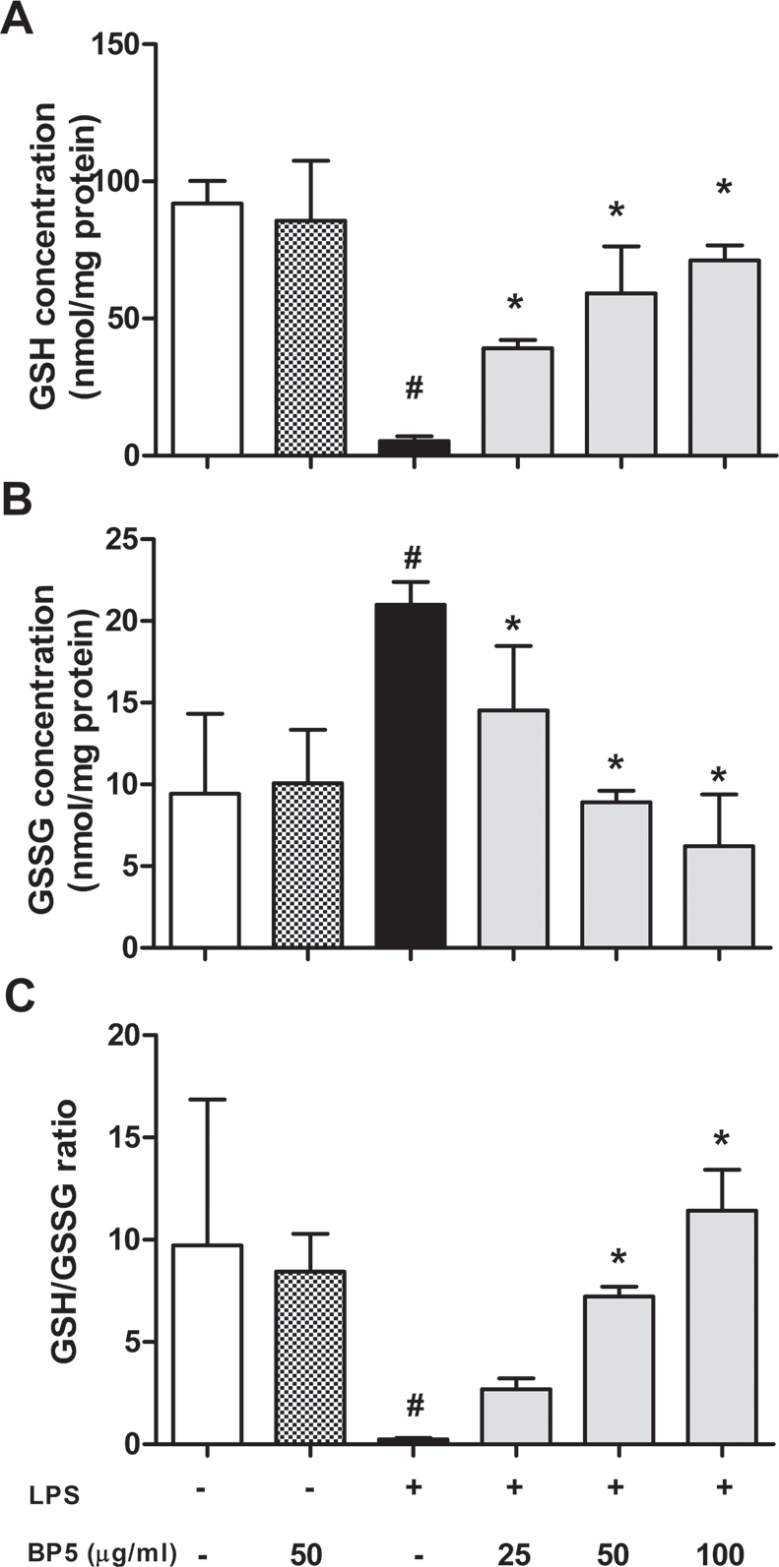
BP5 modulated intracellular GSH, GSSG and the GSH/GSSG ratio in the LPS-stimulated DCs. DCs (1×10^6^ cells/ml) were pretreated with or without BP5. After 2 h, the cells were stimulated with LPS (100 ng/ml) or not for 22 h. The levels of (A) GSH, (B) GSSG and (C) GSH/GSSG ratio in the cells were measured as described in Materials and Methods. Data shown are the means ± SD of three samples. *P < 0.05 in comparison with the LPS-only group; ^#^ *P* < 0.05 in comparison with the untreated group. All results are representative of three independent experiments.

Glutathione emerges as the main defense for the maintenance of the appropriate redox environment [42]. We next studied the modulation of the reduced glutathione (GSH), the oxidized glutathione (GSSG) and their ratio (GSH/GSSG) by BP5 in the LPS-stimulated DCs. In the only-LPS group, GSH (Fig. 4A) was decreased significantly, while GSSG (Fig. 4B) was significantly increased in contrast with control. However, the GSH and GSSG levels were reversed by BP5 (25–100 μg/ml) pretreatment. Besides, an important reduction can be observed with the GSH/GSSG ratio (Fig. 4C).

### BP5 enhanced antioxidant enzymes activities in the LPS-stimulated DCs

**Fig 5:**
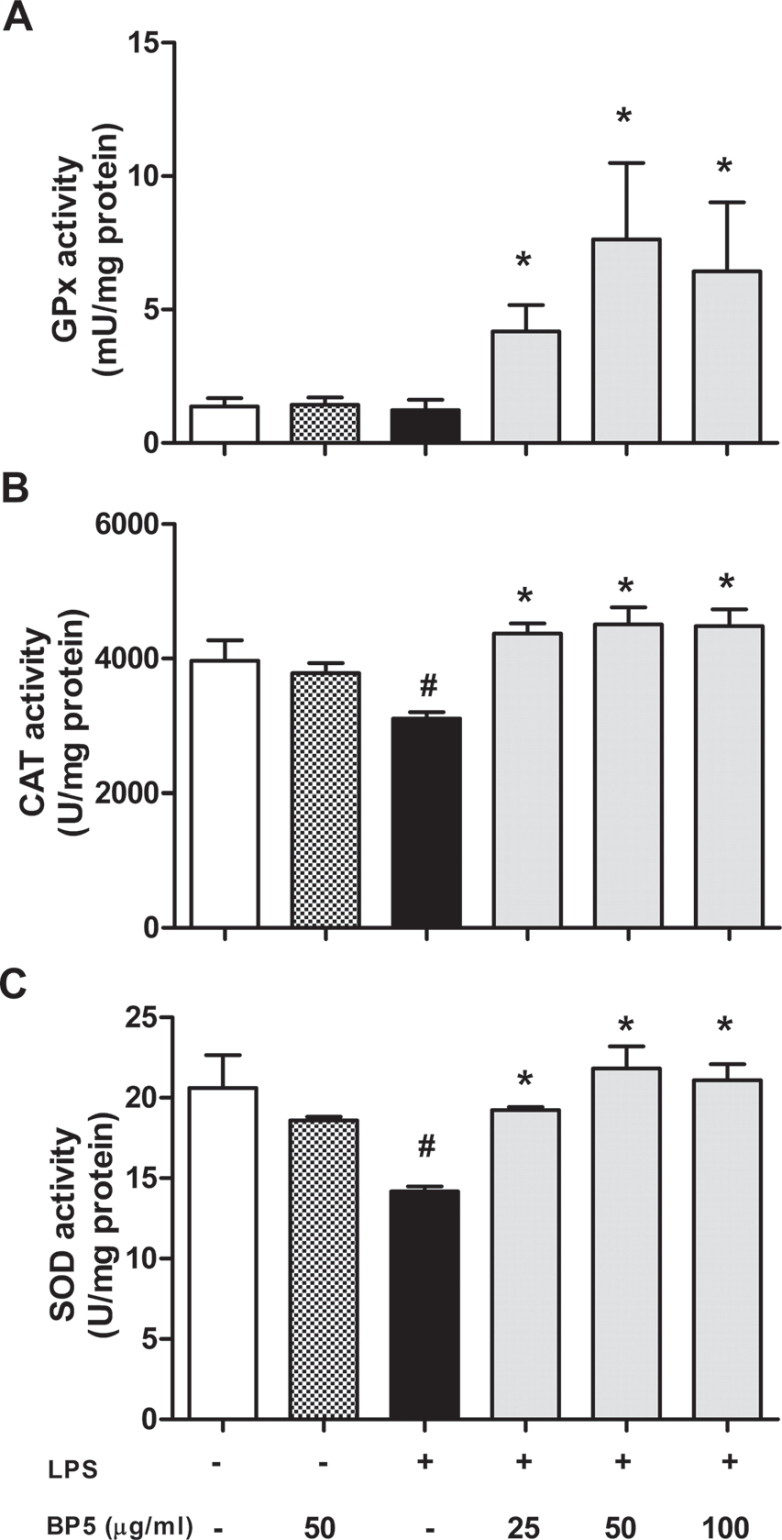
BP5 enhanced the activities of antioxidant enzymes in the LPS-treated DCs. DCs (1×10^6^ cells/ml) were pretreated with BP5 for 2 h, followed by stimulation with or without LPS (100 ng/ml). After 22 h, Intracellular levels of (A) GPx, (B) CAT and (C) SOD were measured using commercial kits. Data shown are the means ± SD of three samples. *P < 0.05 in comparison with the LPS-only group; ^#^ *P* < 0.05 in comparison with the untreated group. All results are representative of three independent experiments.

Previous studies have shown that cell is well equipped with defense mechanisms against oxidative stress-induced cell damage, including antioxidant enzymes such as GPx, CAT and SOD [43]. Here, we have examined the effects of BP5 on the activities of GPx, CAT and SOD in the LPS-activated DCs. When treated with LPS alone, the activities of CAT (Fig. 5B) and SOD (Fig. 5C) in DCs were decreased significantly, suggesting that the anti-oxidant system of DCs was disturbed. As expected, BP5 significantly preserved the activities of the GPx, CAT and SOD (Fig. 5A, B and C).

### BP5 suppressed the maturation of submucosal DCs in the LPS-stimulated ECs/DCs coculture system

**Fig 6:**
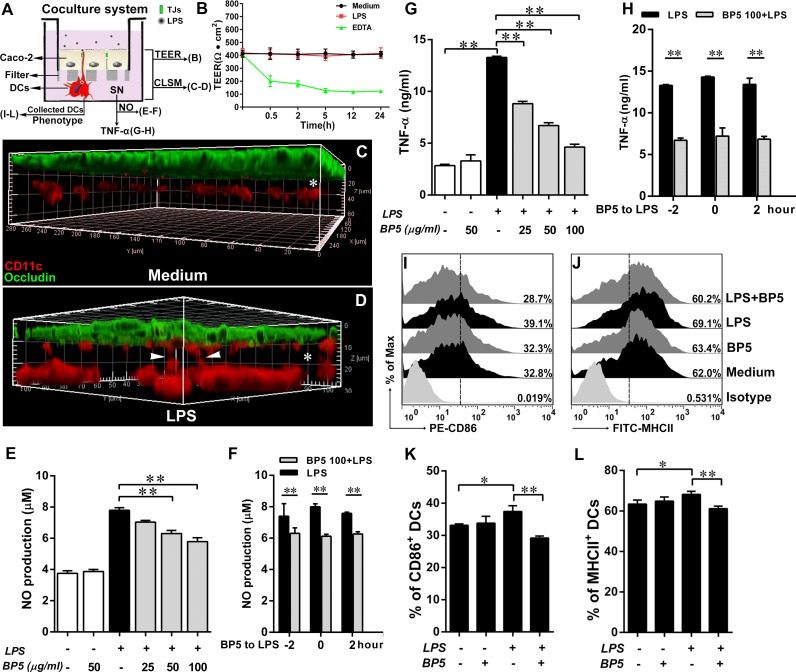
BP5 suppressed the DC maturation in the LPS-stimulated ECs/DCs coculture system. Experimental setting to study the DC maturation in the ECs/DCs coculture system. (A) The scheme depicts: Caco-2 cells were grown on the filter to form a tight monolayer, and then DCs were cultured facing the basolateral side of Caco-2 cells on the bottom of the filter for 4 h. Pretreatment of BP5 on the basolateral DCs for 2 h, medium or LPS (300 ng/ml) was incubated on the apical side of the ECs for 22 h, then the basolateral DCs and culture supernatants were collected. (B) TEER was measured by a Millicell-ERS epithelial voltohmmeter (Millipore) at indicated time. (C-D) The filters were fixed with 4% paraformaldehyde and then processed to immunofluorescence stain for CLSM. Three-dimensional rendering of representative fields was obtained with Imaris 7.2 software, DCs (red, CD11c), tight junction of ECs (green, occludin). Submucosal DCs sent dendrites (arrows) to creep through ECs in response to LPS but not medium. (E-F) Supernatants were collected and NO production was measured using the Griess reagent. (G-H) TNF-α released from basolateral supernatants was measured by ELISA. (F, H) DCs stimulated with LPS before, at the same time as, or after BP5 (100 μg/ml) incubation. (I-L) The expressions of CD86 and MHCII on DCs were analyzed by FACS. Data shown are the means ± SD of three samples. *P < 0.05; **P < 0.01. SN, supernatants. To confirm the results, we repeated these experiments three times.

Endotoxin-LPS also played a vital role in intestinal injury, especially for oxidative and inflammatory injury [44]. And submucosal DCs are the most important participators for triggering the inflammatory response of intestinal mucosa [45]. Thus, we established a coculture system containing Caco-2 intestinal epithelial cells (ECs) and DCs *in vitro* (Fig. 6A). This system allowed simplifying the mucosal barrier to just 3 players: DCs, ECs, and LPS in a spatial distribution similar to that found *in vivo*. Firstly, we confirmed that LPS had a powerful ability to induce submucosal DCs to send their dendrites across the ECs (Fig. 6C-D), without disrupting ECs barrier (Fig. 6B), suggesting that transepithelial dendrites were the bridges between LPS and submucosal DCs, and were essential for subsequent DC maturation. As expected, incubation with LPS on the apical side of ECs in the coculture system, the production of NO (Fig. 6E) and TNF-α (Fig. 6G) from basolateral supernatants were also remarkably increased. Similarly, CD86 and MHCII, the phenotypic markers of DCs, were significantly upregulated compared with that treated with medium only (Fig. 6I-L). However, they were significantly inhibited by pretreatment of submucosal DCs with BP5. Of note, NO (Fig. 6F) and TNF-α (Fig. 6H) were decreased in all tests, regardless of whether the BP5 treatment was incubated to DCs before, at the same time as, or after LPS stimulation. These data suggested that BP5 attenuated submucosal DC maturation in the LPS-stimulated ECs/DCs coculture system.

### HO-1 was upregulated by BP5 in the LPS-stimulated DCs and played a key role in suppression of oxidative stress and DC maturation

**Fig 7:**
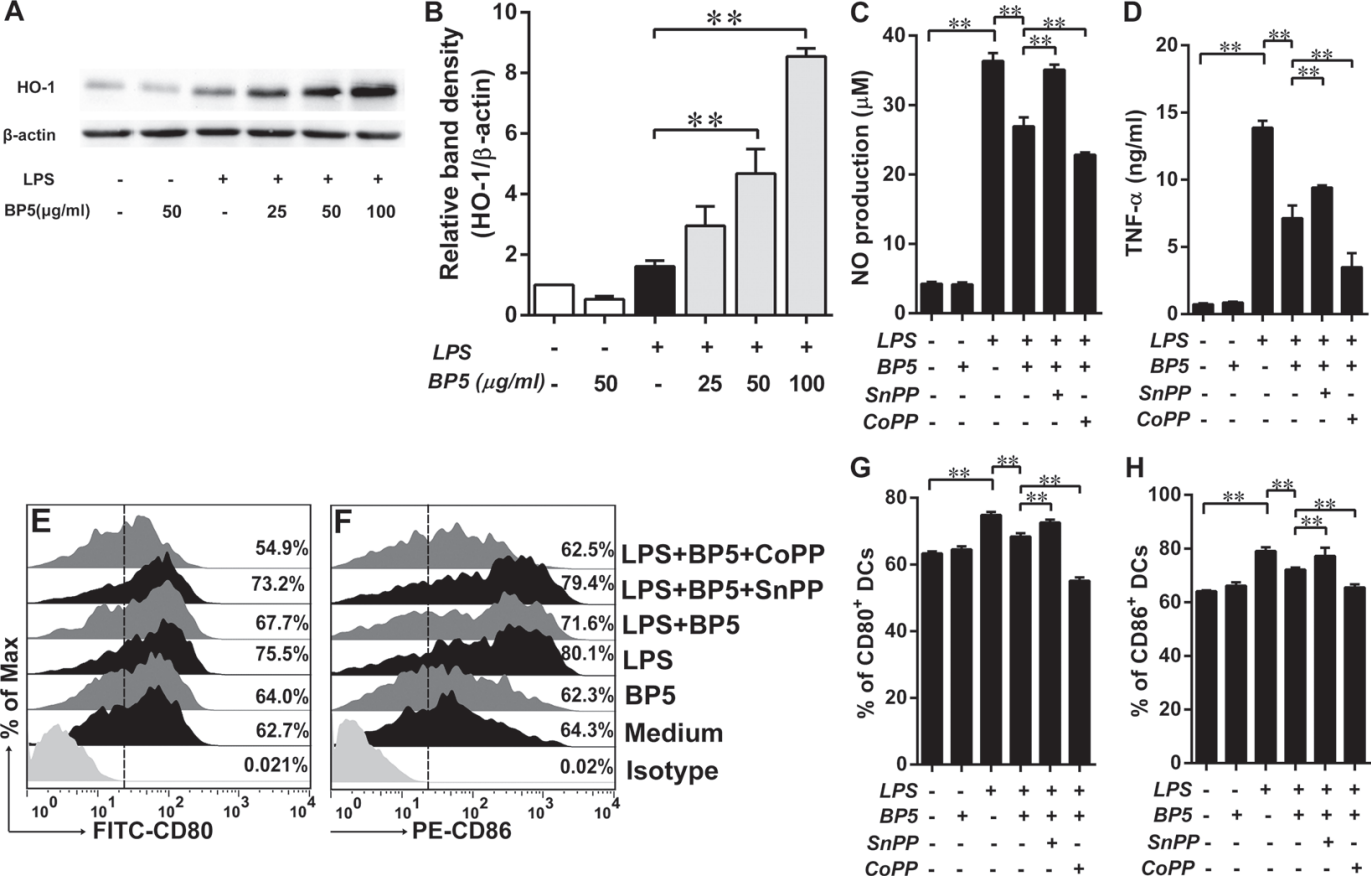
BP5 upregulated the expression of HO-1 protein in the LPS-induced DCs. DCs (1×10^6^ cells/ml) were treated with the indicated concentrations of BP5 for 2 h, and then incubated with or without LPS (100 ng/ml) for 22 h. (A) HO-1 levels were assessed by western blotting. (B) Quantification of the blots. (C-H) DCs were pretreatment with BP5 (100 μg/ml) in the presence or absence of Snpp (25 μM) or Copp (50 μM) for 2 h, and then incubated with or without LPS (100 ng/ml) for 22 h. (C) Supernatants were collected and NO production was measured using the Griess reagent. (D) TNF-α released from supernatants was measured by ELISA. DCs were harvested and the expressions of CD80 (E, G) and CD86 (F, H) were analyzed by FACS. Data shown are the means ± SD of three samples. *P < 0.05; **P < 0.01. All results are representative of three independent experiments.

Recent studies have implied that antioxidative and anti-inflammatory activations were closely associated with the induction of HO-1, a microsomal enzyme involved in the rate-limiting step in the degradation of heme to biliverdin [46–49]. We next investigated whether BP5 increased HO-1 expression in the LPS-stimulated DCs. Our results demonstrated that, pretreatment of LPS-activated DCs with BP5, HO-1 was remarkably upregulated, compared with LPS-only group (Fig. 7A, B). Our current study in coculture system and previous study in monoculture [37] proved that BP5 can attenuate the maturation of DCs. Next, to determine whether HO-1, induced by BP5, played an important role in the suppression of oxidative stress and DC maturation, we detected the NO production (Fig. 7C), proinflammatory cytokine (TNF-α, Fig. 7D) release and phenotypic maturation (CD80 and CD86, Fig. 7E-H). Of note, SnPP, an inhibitor of HO-1 activity, reversed the effect of BP5 on the LPS-stimulated DCs. However, CoPP, an inducer of HO-1, aggravated the inhibitory effect of BP5 on the LPS-stimulated DCs (Fig. 7C-H).

## Discussion

Here, we explored the antioxidative effects of BP5 on DCs in the presence and absence of intestinal epithelial barriers. Together, these analyses demonstrate the potent immunosuppressive activity of BP5 on DCs. BP5 was shown to reduce NO production, intracellular ROS, and lipid peroxidation in LPS-induced DCs, along with restoring depleted glutathione levels in terms of the GSH/GSSG ratio. Meanwhile, the activities of various antioxidant enzymes (GPx, CAT, and SOD) were enhanced during the above processes. BP5 suppressed the maturation of submucosal DCs in the LPS-stimulated EC/DC coculture system. Importantly, BP5 was capable of enhancing HO-1 expression in LPS-stimulated DCs, and HO-1 plays a vital role in the suppression of oxidative stress and DC maturation.

The molecular structure of BP5 contains a thiol group, a key antioxidative component, within the cysteine residue, implying that the natural role of BP5 may be as an antioxidant. A similar structure, glutathione, contains an active thiol group, which confers protective activity against a range of cellular stresses, such as toxins and oxidative stress [50]. Another naturally occurring peptide, N-acetylcysteine (NAC), also acts as a thiol-containing antioxidant.

Excess NO can react with O_2_^−^ to form ONOO^−^, resulting in oxidative stress and cell damage [51]; high level ROS production also induces significant oxidative stress, resulting in lipid peroxidation and loss of cellular function, followed by apoptosis or necrosis [52,53]. The data presented here suggest that BP5 impaired the production of NO, ROS, and lipid peroxidation in LPS-induced DCs.

Evidence of reduced oxidative stress led us to investigate potential mechanisms underlying the antioxidative effects of BP5 in DCs. In traditional cellular oxygen-scavenging systems, glutathione redox status and antioxidant enzymes play important roles. As the mitochondrial electron transport chain is a major source of cellular ROS, retention of GSH by mitochondria may represent a critical defence against ROS damage [54,55], with depletion of mitochondrial GSH leading to increased ROS formation [56]. In many cases, GSH can convert itself to GSSG in response to oxidative stress; however, in this reaction, the reduction of hydrogen peroxide is catalysed by the GSH-Px enzyme [42]. In the present study, our results confirmed that of previous research demonstrating an LPS-induced decrease in the GSH/GSSG ratio in DCs [57], peritoneal macrophages [58], lymphocytes [58], and other different models of septic shock [59]. Crucially, the addition of BP5 to these cells dramatically attenuated these effects, indicative of an increase in GSH levels, the GSH/GSSG ratio, and GPx enzyme activity, along with additional decreases in the intracellular oxidative stress of DCs.

Under normal conditions, SOD function as the first stage of antioxidant defence by catalysing O_2_- into H_2_O_2_ and O_2_, while CAT also contributes to the breakdown of H_2_O_2_ [60]. Considering the various types of oxidative thiol modifications that may affect antioxidant enzyme activities, especially under conditions of oxidative stress [61], we directly measured the activities of CAT and SOD. Our results indicated that BP5 was able to enhance the activities of CAT and SOD in the LPS-induced DCs, suggesting that a high GSH/GSSG ratio along with increased antioxidant enzyme activities might be beneficial to the scavenging of ROS.

Under certain conditions, NO has been shown to induce the secretion of proinflammatory cytokines, including TNF-α and IFN-γ [62,63]. Similarly, ROS is involved not only in the activation and maturation process of DCs [64], but also acts as an important regulator of DC-T cell communication during antigen presentation [65], while lipid peroxidation acts as a major component of chronic inflammation [66]. Oxidative stress is therefore broadly associated with immune activation and the inflammatory response. The therapeutic use of antioxidants, such as glutathione and NAC, for the treatment of chronic inflammatory diseases and cancers [67,68] inspired us to further explore the use of BP5 as a potential treatment of inflammatory diseases. Preliminary studies of BP5 in LPS-induced DC activation strongly supported this observation, indicating a potential role for BP5 as a therapeutic agent [37].

In the complex intestinal environment, an increase in gram-negative bacteria has been observed in intestinal inflammatory diseases, including inflammatory bowel disease [69]. One possible explanation for this effect may be that the elevated LPS levels associated with an increase in gram-negative bacteria may overstimulate the immune system, resulting in chronic intestinal inflammation [70]. Our results, along with that of a previous study [71], are consistent with a model of increased LPS causing submucosal DCs to send their dendrites across the intestinal epithelial barrier. Such a result implies that LPS could be in direct contact with the submucosal DCs, triggering a DC-mediated inflammatory response. Evidence for such a hypothesis can be seen in our EC/DC coculture analyses in which LPS activated the maturation of submucosal DCs, even in the presence of an epithelial barrier. As in our previous monoculture experiments [37], BP5 also had a strong inhibitory effect, suggesting that BP5 may represent a promising candidate for the treatment or prevention of intestinal inflammatory diseases.

HO-1 is a stress inducible enzyme that catalyses the degradation of haem proteins into free iron, carbon monoxide (CO), and biliverdin (BV), which is then converted rapidly into bilirubin (BR). These catabolic end products exert antioxidative and antiapoptotic properties, rendering the overall function of HO-1 to be cytoprotective [72]. Upregulation of HO-1 reduced the expression of inducible nitric oxide synthase (iNOS), resulting in an attenuation of NO production [73], while HO-1 deficient cells exhibited increased intracellular ROS generation, suggesting that a certain level of HO-1 expression could be required for the scavenging of ROS [74]. Like other oxidative stress response enzymes, HO-1 exerted both immunomodulatory and anti-inflammatory functions [47]. HO-1 end products, such as CO, can negatively regulate toll-like receptor signalling [75], while BR impairs MHCII expression in endothelial cells [75]. Furthermore, HO-1 activation can inhibit the function of both T and B cells, and its deficiency is related to the development of chronic inflammation [75]. In terms of DC function, HO-1 has been identified as a potential regulator of DC maturation via the p38 MAPK pathway [76]. Here, we have demonstrated that BP5 inhibited the maturation of LPS-induced DCs via the induction of HO-1 expression. These results are similar to that of a microarray analysis performed using mouse-derived hybridoma cells, which demonstrated *HMOX1* upregulation in response to BP5 [35].

In summary, the current study demonstrates a clear antioxidative role for BP5 in the protection of LPS-induced DCs against oxidative stress, significantly attenuating their immune function in the presence of intestinal epithelial barriers. These analyses suggest a possible role for BP5 in the treatment and prevention of a variety of chronic inflammatory and autoimmunity diseases.
